# Lightweight Deep Neural Network Method for Water Body Extraction from High-Resolution Remote Sensing Images with Multisensors

**DOI:** 10.3390/s21217397

**Published:** 2021-11-07

**Authors:** Yanjun Wang, Shaochun Li, Yunhao Lin, Mengjie Wang

**Affiliations:** 1 Hunan Provincial Key Laboratory of Geo-Information Engineering in Surveying, Mapping and Remote Sensing, Hunan University of Science and Technology, Xiangtan 411201, China; lsc_gis@mail.hnust.edu.cn (S.L.); linyunhao@mail.hnust.edu.cn (Y.L.); wangmengjie@mail.hnust.edu.cn (M.W.); 2 National-Local Joint Engineering Laboratory of Geo-Spatial Information Technology, Hunan University of Science and Technology, Xiangtan 411201, China; 3 School of Resource Environment and Safety Engineering, Hunan University of Science and Technology, Xiangtan 411201, China

**Keywords:** water body extraction, multisensor high-resolution image, lightweight deep neural network, MobileNetv2, deep learning

## Abstract

Rapid and accurate extraction of water bodies from high-spatial-resolution remote sensing images is of great value for water resource management, water quality monitoring and natural disaster emergency response. For traditional water body extraction methods, it is difficult to select image texture and features, the shadows of buildings and other ground objects are in the same spectrum as water bodies, the existing deep convolutional neural network is difficult to train, the consumption of computing resources is large, and the methods cannot meet real-time requirements. In this paper, a water body extraction method based on lightweight MobileNetV2 is proposed and applied to multisensor high-resolution remote sensing images, such as GF-2, WorldView-2 and UAV orthoimages. This method was validated in two typical complex geographical scenes: water bodies for farmland irrigation, which have a broken shape and long and narrow area and are surrounded by many buildings in towns and villages; and water bodies in mountainous areas, which have undulating topography, vegetation coverage and mountain shadows all over. The results were compared with those of the support vector machine, random forest and U-Net models and also verified by generalization tests and the influence of spatial resolution changes. First, the results show that the F1-score and Kappa coefficients of the MobileNetV2 model extracting water bodies from three different high-resolution images were 0.75 and 0.72 for GF-2, 0.86 and 0.85 for Worldview-2 and 0.98 and 0.98 for UAV, respectively, which are higher than those of traditional machine learning models and U-Net. Second, the training time, number of parameters and calculation amount of the MobileNetV2 model were much lower than those of the U-Net model, which greatly improves the water body extraction efficiency. Third, in other more complex surface areas, the MobileNetV2 model still maintained relatively high accuracy of water body extraction. Finally, we tested the effects of multisensor models and found that training with lower and higher spatial resolution images combined can be beneficial, but that using just lower resolution imagery is ineffective. This study provides a reference for the efficient automation of water body classification and extraction under complex geographical environment conditions and can be extended to water resource investigation, management and planning.

## 1. Introduction

Water is the source of life and primary factor for maintaining the sustainable development of the earth’s ecological environment, and it has an important impact on public health, living environment and economic development [1]. Therefore, timely and accurate large-scale regional water body surveys and dynamic monitoring are of great significance for water resource planning, flood control and disaster reduction [2]. In recent years, satellite remote sensing technology has developed rapidly [3]. As an important method of obtaining water body information, remote sensing images have been widely used in mapping, geography, environmental protection, military reconnaissance and other fields [4]. Most of the previous water resource surveys were based on medium- and low-resolution remote sensing images [5], and their limited spatial resolution made it difficult to extract small-area water bodies, such as broken lakes and slender rivers, in complex areas [6]. With the successful launch of high-resolution optical satellites (such as Worldview-2 and GF-2), the spatial resolution of satellite remote sensing images has also been improved from the meter level to the submeter level [7]. High-resolution remote sensing images have more detailed spatial, texture, geometry and other ground feature information [8] and thus can more clearly differentiate water bodies in complex scenes. However, optical satellite remote sensing can also produce low-quality images caused by cloud cover in bad weather and long return periods, which makes it difficult to collect and process real-time data [9]. Unmanned aerial vehicle (UAV) remote sensing can overcome the above limitations [10], and the resolution of UAV orthophoto images reaches the centimeter level; thus, the images present extremely rich ground object details [11]. At present, an increasing number of scholars use high-resolution optical satellite remote sensing images and UAV orthophotos to extract surface water [12].

Water body extraction methods can be mainly divided into (1) extraction methods based on image spectral characteristics (single-band threshold method, multiband spectral relationship method, water index method) [13,14,15]; (2) classifier methods (object-oriented method, decision tree method, support vector machine (SVM) method, random forest (RF) method, etc.) [16,17,18,19]; and (3) deep-learning methods [20,21,22].

(1) The traditional water index method based on image spectral characteristics is widely used in the research field of water body extraction because of its fast calculation speed, high precision and wide implementation range [23]. McFeeters et al. [24] constructed a normalized differential water index (NDWI) by using green band and near-infrared band in TM image, which can suppress vegetation information to the maximum extent and highlight water information. However, because water body and shadow have similar spectral characteristics, and the reflected signals of buildings and soil in urban areas are stronger than water body, soil, buildings and shadows are confused with water body. Xu et al. [25] constructed a modified normalized differential water index (MNDWI) by replacing the near-infrared band in NDWI with the mid-infrared band, which can effectively reduce the influence of buildings and soil on water extraction, but there is still the interference of shadow information on water. The water index method has great differences in the optimal thresholds on different occasions, and the selection of the optimal threshold needs to be determined according to research experience [26]. Therefore, this method is subjective and low in automation [27]. Although new algorithms have been proposed to alleviate the above difficulties [28,29], these improved methods are still concentrated on the calculation of spectral information of remote sensing images and do not consider the spatial and texture characteristics of images [30]. Moreover, these methods cannot be fully used for high-spatial-resolution remote sensing images with only four bands ( red, green, blue and near-infrared), which seriously restricts the detection ability of small areas or narrow water bodies in complex regions [31]. In the case of high-spatial-resolution remote sensing images, the widespread shadow problem is more complicated due to high urban buildings and mountain vegetation [32].

(2) Considering the above difficulties, Li et al. [33] combined the statistical characteristics of spectral information of image segmentation and the shape characteristics of shadow removal to extract water bodies from high-resolution images at different levels. The results improved the extraction accuracy of water bodies from high-resolution images and reduced the influence of shadows. Although this method considers the shape characteristics of high-resolution images, the degree of automation is still low, and it needs to be combined with the supervised classification method. Huang et al. [34] first calculated the water, shadow and vegetation indices as the initial pixel level results and then combined them with an object-based machine learning method applied to water-type recognition in GeoEye-1 and WorldView-2 high-resolution images. The results showed that the decision tree method using texture and geometric features is far better than other machine learning classification methods and water index methods and greatly improves the automation of water body recognition and further reduces the impact of shadows in urban areas. The classifier method removes a portion of the influence on water bodies of similar spectral features, such as shadows and buildings, although its feature extraction and classifier design are cumbersome. The results are excessively dependent on the selection of limited sample sets [35] and cannot deeply mine the information characteristics of ground objects, which results in insufficient generalization ability, and different seasons and images are not universal [36].

(3) With the development of deep learning technology, end-to-end convolutional neural networks (CNNs) show great advantages in extracting the spatial context relationship of images [37] and can automatically extract more abstract high-dimensional features from the input low-level image features [38]. Currently, various new deep learning models have been applied to the classification and change detection of ground objects in high-resolution remote sensing images, such as VGGNet [39], FCN (full convolution network) [40], ResNet [41] and U-Net [42]. Compared with the machine learning classification method, even if the initial sample size is insufficient, the deep learning method can also enrich the sample size through data expansion [43] to achieve higher extraction accuracy. For example, Li et al. [44] used the FCN model to extract water bodies from GF-2 high-resolution remote sensing images under the condition of limited training samples, and the results were significantly better than those of the NDWI and SVM methods. Li et al. [45] combined the two visual features of the gray level co-occurrence matrix (GLCM) [46] and Gabor filtering and input them into the improved U-Net to extract the water body of UAV high-resolution remote sensing images. The results showed the stability and accuracy of the improved U-Net method combined with GLCM features. The successive proposal of deep learning networks has continuously improved the accuracy of classification tasks and increased the network performance. However, the layers of deep neural networks are also deepening and the structure is becoming increasingly complex [47], which will lead to the rapid increase in the number of model parameters and the consumption of computational resources, thereby complicating model training and hindering model application in actual demand scenarios [48]. Accordingly, Howard et al. [49] proposed MobileNetV1 in 2017, which uses deep separable convolution to construct lightweight deep neural networks, and it can not only reduce the number of parameters and computation but also maintain high image classification accuracy. Subsequently, the proposed MobileNetV2 [50] further improved the performance of the network by using the reverse residual structure. At present, water body surveys require lightweight networks with high efficiency, high convenience and high accuracy in the field of intelligent extraction of high-resolution remote sensing images.

In summary, the traditional method of extracting water bodies from high-spatial-resolution remote sensing images easily identifies shadows and buildings as water bodies, cannot easily extract small water bodies and mountainous water bodies, and presents weak generalization ability. The existing deep convolutional neural network model has a long training time, and the computational resources requirements are high; thus, it cannot meet real-time requirements. In this paper, the lightweight network MobileNetV2 was used to extract the water bodies in complex scenes from high-resolution remote sensing images of GF-2, Worldview-2 and UAV orthophotos with three different sensors, and the extraction, accuracy evaluation and model efficiency results of SVM, RF and U-Net were compared. The purpose was to select an efficient, convenient and accurate water body extraction method under complex geographical conditions based on high-resolution remote sensing images and to provide a reference for the real-time and rapid extraction of water distribution information and rational utilization of water resources.

## 2. Study Area and Data

### 2.1. Study Area

**Figure 1:**
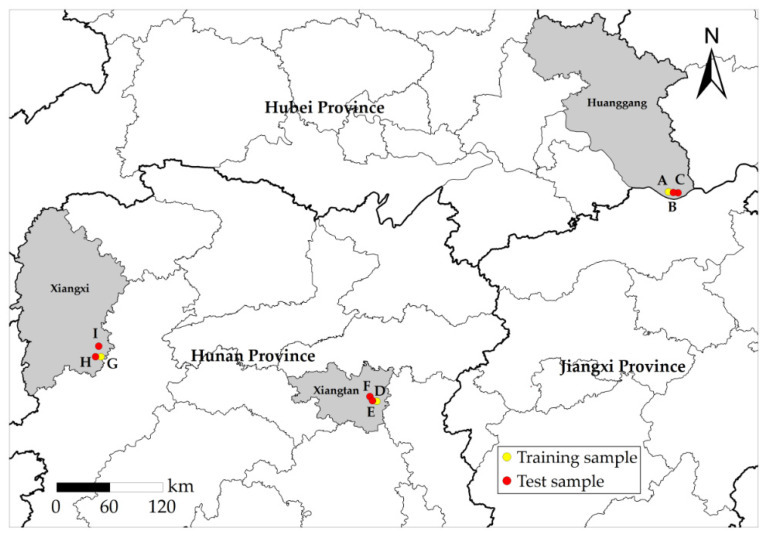
Study area and sample distribution. Area *A*–*C* images were obtained by GF-2, and their longitudes and latitudes range from 115°56′20″ E, 29°49′02″ N to 116°00′32″ E, 29°45′22″ N. Area *D*–*F* images were obtained by Worldview-2, and their longitudes and latitudes range from 112°46′09″ E, 27°39′55″ N to 112°48′34″ E, 27°38′06″ N. Area *G*–*I* images were obtained by UAV, and their longitudes and latitudes range from 110°05′30″ E, 28°07′36″ N to 110°06′36″ E, 28°06′52″ N. Area *A*, *D* and *G* samples were used for model training. Area *B*, *E*, *H* samples were used for model testing. Samples from areas *C*, *F*, and *I* were used for model generalization verification.

To explore the applicability of different sensors for different types of water bodies, GF-2, Worldview-2 and UAV orthophoto images were selected as experimental data, and they correspond to Huangmei County, Xiangtan County and Luxi County in China, respectively. Huangmei County is located on the northern bank of the middle reaches of the Yangtze River. The water bodies in this region are vast and include numerous rivers, lakes and reservoirs, and ponds and weirs are densely distributed. Xiangtan County is located on the south bank of the upper reaches of the Xiangjiang River. The region is rich in water resources, and the total amount of surface water resources is 603.75 billion kilometers. Luxi County is located on the southwest bank of the Yuanjiang River and has mountainous terrain, many waters bodies and a basin area of 1565.5 km^2^. The study area of this experiment is shown in Figure 1.

The types of surrounding water bodies in the three study areas are different. There are many asymmetrically distributed, broken-shaped and slender farmland irrigation water bodies in Huangmei County (areas *A*–*C*) and Xiangtan County (areas *D–F*), such as ditches and ponds, while a large area of water bodies and some small tributaries are found in Luxi County (areas *G*–*I*). In addition, the study areas contain a large number of ground feature elements, such as farmland, township buildings, vegetation, mountains and shadows of various elements. The shape characteristics and spectral characteristics of these ground feature elements are similar to those of water, which poses challenges to water body extraction.

### 2.2. Data Source and Preprocessing

**Figure 2:**
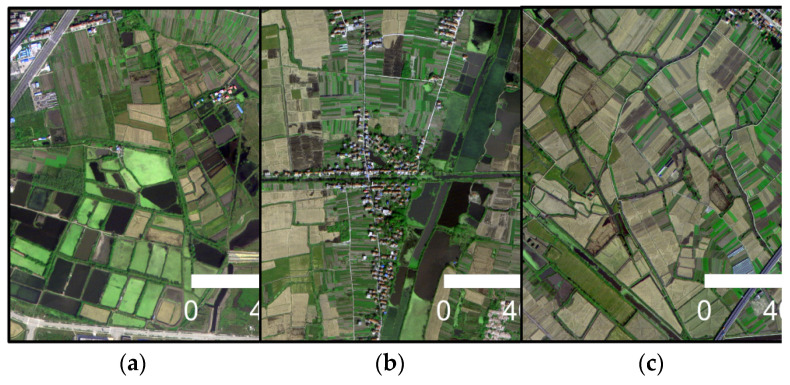
Different types of water bodies obtained by GF-2. (**a**) Broken lake surface; (**b**) dark lake; and (**c**) slender ditches.

**Figure 3:**
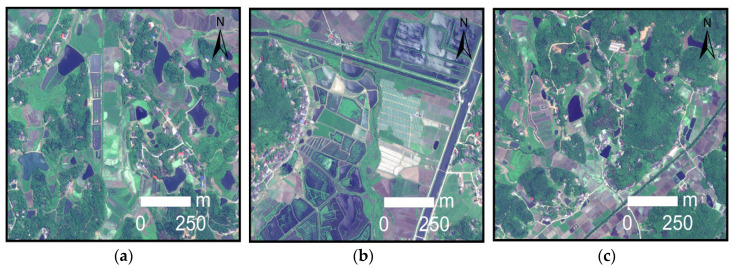
Different types of water bodies obtained by Worldview-2. (**a**) Irrigation lakes; (**b**) duckweed lakes; and (**c**) trenches.

**Figure 4:**
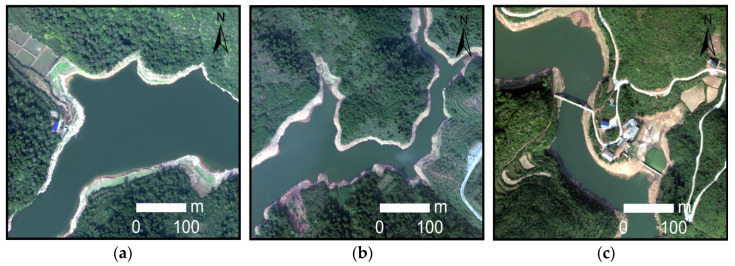
Different types of water bodies obtained by UAV. (**a**) Wide lake surface; (**b**) curved lake; and (**c**) artificial lake.

The specific parameters of GF-2, WorldView-2 and UAV images obtained in this study are shown in Table 1. The GF-2 remote sensing satellite is a high-resolution optical remote sensing satellite launched by China on 19 August 2014. It has a 1-m spatial resolution panchromatic band and four 4-m spatial resolution multi-spectral bands (red, green, blue and near-infrared). Launched on 6 October 2009, WorldView-2 is the world’s first high-resolution 8-band multi-spectral commercial satellite, providing panchromatic images with a spatial resolution of 0.5 m and multi-spectral images with a spatial resolution of 1.8 m (including 4 standard bands: red, green, blue and near-infrared, and 4 additional bands: coast blue, yellow, red rim and nearinfrared 2). Unmanned aerial vehicle remote sensing has become one of the most important methods for acquiring image data in recent years. Compared with satellite images, it has the advantages of a low cost, fast transmission speed and is not limited by geographical environment [51]. However, UAV remote sensing also has the problems of a small load, short endurance time, small image coverage and low productivity. Therefore, making good use of the advantages of different high-resolution sensors is very important for the extraction of a large range of complex water bodies. In this study, the remote sensing image of areas *A*–*C* were derived from GF-2, and the remote sensing image of areas *D*–*F* were derived from WorldView-2. The high-resolution remote sensing image data of GF-2 and WorldView-2 are processed by image fusion and band combination in ENVI 5.3 software, and the output is RGB image. Firstly, the panchromatic band and multi-spectral band of GF-2 and WorldView-2 images were processed by Gram–Schmidt image, and 4-band GF-2 image data with spatial resolution of 1 m and 8-band WorldView-2 image data with a spatial resolution of 0.5 m were obtained. Then, the RGB bands of the fused GF-2 image and WorldView-2 image were selected for band combination, and the output image was a GeoTIFF format. Remote sensing images of areas *G*–*I* were obtained with UAV remote sensing technology. It used a six-rotor UAV (KPM-28, Hunan Kunpeng Zhihui UAV Technology Co., LTD., Changsha, China) equipped with SHARE-101S tilt camera (Shenzhen Pengjin Technology Co., LTD., Shenzhen, China). The camera contains five complementary metal oxide semiconductor (CMOS) sensors (23.5 mm×15.6 mm) with 24.3 million effective pixels and a 35 mm focal length. Since the coverage of images acquired by UAV flight is small, aerial images acquired in different areas need to be spliced in Pix4D and then encrypted in three-dimensional space, so the measured ground control points were used to match the location information of UAV images to prevent geometric distortion. After orthography correction was completed, the UAV image was output to the visible light red, green and blue (RGB) mode with a spatial resolution of 0.2 m. In this study, after radiometric calibration and geometric correction of the three-view images obtained, GF-2 images were cropped to 10,080×10,080 image size, and WorldView-2 and UAV images were cropped to 13,440×10,080 image size, which were used as the construction range of water sample database of high-resolution remote sensing images. The three-view images are mainly the shadows, township building areas and mountainous areas containing more complex water types, and come from different seasons, different periods and different shooting angles. Figure 2, Figure 3 and Figure 4 shows typical complex water bodies in GF-2, WorldView-2 and UAV images respectively.

## 3. Methods

**Figure 5:**
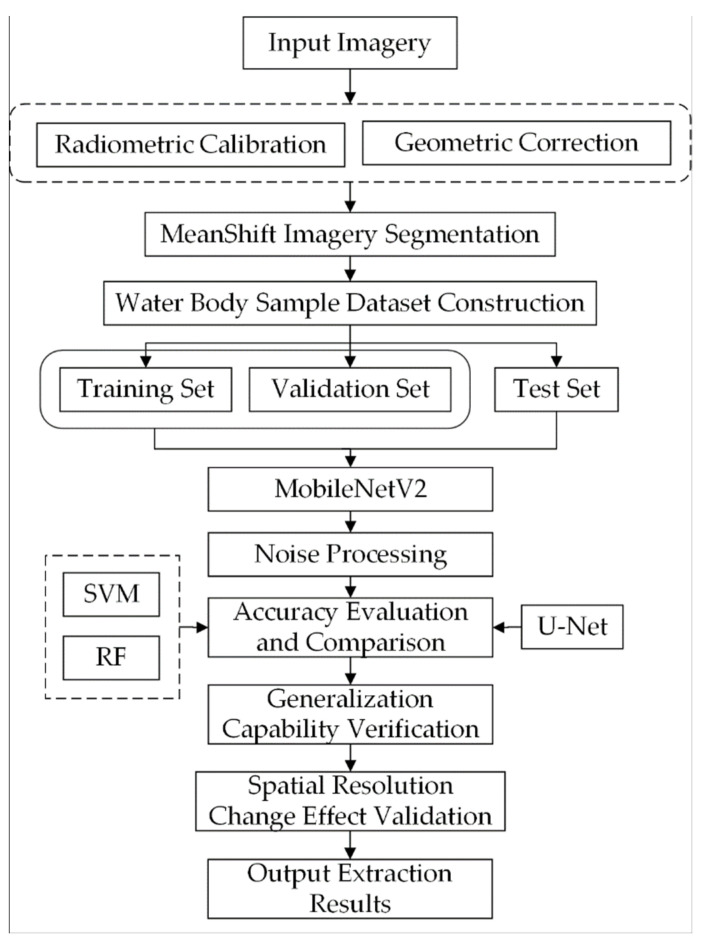
Experimental flow chart applied separately to each of the 3 sets of input imagery: GF-2, Worldview-2, and UAV. In Section 5.3, the MobileNetV2 model tests various combinations of the input imagery.

In this work, a lightweight deep neural network, MobileNetV2, was applied to extract water bodies from complex scenes in high-resolution remote sensing images obtained by 3 different sensors. All experimental flows in this study are shown in Figure 5. Firstly, the images were corrected and clipped, including radiometric correction, geometric correction and clipping of the study area, to generate high-resolution RGB image data with obvious features and accurate positions. Then the cropped images were segmented by Mean Shift, which combines the adjacent pixels with similar spectral, texture and shape features together, which is more conducive to the interpretation of different ground objects by human eyes. After image data preprocessing, the water and non-water samples in the high-resolution image were manually vector labeled in ArcGIS10.6 to construct the water extraction sample set of the high-resolution remote sensing image, in which the images and vector labeled results of areas *A*, *D* and *G* were used as the training and verification sample data set. The image and vector annotation results of areas *B*, *E* and *H* were used as the test sample data set, and the image and vector annotation results of area *C*, *F* and *I* were used as the generalization test sample data set. The proportion of the 3 sample data sets was 6:2:2, respectively. Next, we introduced the MobileNetV2 model into pixel classification, and input the training sample data sets of 3 different sensors into the deep learning model for individual training. The corresponding input of the trained model was put into the test images of 3 different sensors for inference, and the classification graph of pixel marking results was output. There were some noises in the result image of pixel classification, and morphological methods were used in this study to remove the noises. Finally, we verified the accuracy of the post-processed classification graph and reference annotation result graph and compare and evaluate the accuracy of MobileNetV2 model with support vector machine and random forest model based on machine learning, and U-Net model based on deep learning. In order to verify the generalization ability of MobileNetV2 model, we further classified and evaluated the accuracy of MobileNetV2 model in more complex generalization test images. In addition, we analyzed the impact of the combination of different sensors and training datasets with different spatial resolutions on the accuracy of the MobileNetV2 model for extracting water bodies.

### 3.1. Machine Learning Water Body Extraction Model for Remote Sensing Images

#### 3.1.1. Support Vector Machine (SVM) Classification Model

The SVM classification model defines a hyperplane in the feature space class to realize the classification of training samples, and it is mainly aimed at the binary classification problem. Even if the statistical sample size is small, this model can also obtain good results [52]. The SVM parameters in this study were selected as radial basis kernel functions.

#### 3.1.2. Random Forest (RF) Classification Model

The random forest classification model is an ensemble learning classification method that combines the bagging ensemble learning theory with the random subspace method. The bootstrap resampling method was used to extract multiple samples from the original sample, and each bootstrap sample was modeled by a decision tree. By combining all the decision trees, the final classification or prediction results were obtained by voting. Compared with other machine learning classification models, the random forest model shows more robust and stronger generalization ability in the classification process and is widely used in land use classification [53]. After several comparative experiments, the maximum number of random forest parameter trees was set as 150 and the maximum depth of trees was set as 90 in this study.

### 3.2. Deep Learning Water Body Extraction Model for Remote Sensing Images

#### 3.2.1. U-Net Model

**Figure 6:**
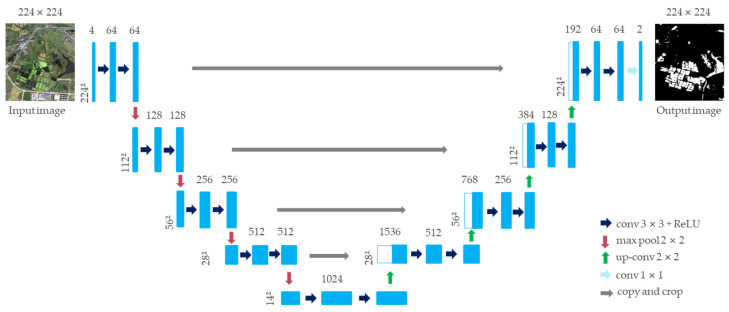
Network architecture of U-Net.

The U-Net network structure is based on the expansion and modification of the fully convolutional neural network (FCN). Its network structure is U-shaped, which is composed of the contraction path used to obtain the context information in the left half and the expansion path symmetrically used to accurately locate the target in the right half, as shown in Figure 6. In the left half of the path, the input image size was 224 subpaths, and the number of dimensions was 4. After passing through f4 convolution layers and pooling layers, a high-dimensional feature map with a size of 14 layers and a number of dimensions of 1024 was obtained. Then, the expansion path of the right half was upsampled layer by layer and fused with the feature map of the corresponding level in the left contraction path. At the last layer of the network, the 1-dimensional convolution layer was used to map the 64-dimensional feature vector to the output image of the 224-feature vector map. Then, the sigmoid function was used for classification, and finally, the water body extraction results were consistent with the spatial resolution of the input image [54].

#### 3.2.2. MobileNetV2 Model

MobileNetV1 is a CNN-based network model. Compared with the traditional CNN model, the proposed model requires less computation and is suitable for working on mobile and embedded devices with low computing power [55]. MobileNetV1 replaces the standard convolution decomposition of the CNN model with a deep separable convolution that contains 2 operations, namely, depthwise convolution and pointwise convolution, replaces the maximum pooling convolution layer with a convolution layer with a step size of 2, and introduces 2 super parameters, namely, a width multiplier and resolution multiplier, to refine the width of the model and reduce the resolution of the input image [56]. These features optimize the network structure size and reduce the computational complexity, so that the network can effectively reduce the number of parameters (*Params*) and the amount of computation (*FLOPs*) while maintaining high image classification accuracy. Assuming that the convolution kernel size is $K_{h}{\times K}_{w}$, the number of input channels is $C_{in}$, the number of output channels is $C_{out}$ and the width and height of the output feature map are W and H, respectively, the complexity calculation formula of the standard convolution layer in the model is as follows [57]:

(1) $${\mathrm{Params}=K}_{h}\times K_{w}\times C_{\mathrm{in}}\times C_{\mathrm{out}}$$

(2) $${\mathrm{FLOPs}=K}_{h}{\times K}_{w}{\times C}_{\mathrm{in}}{\times C}_{\mathrm{out}}\times H\times W=\mathrm{Params}\times H\times W$$

**Figure 7:**
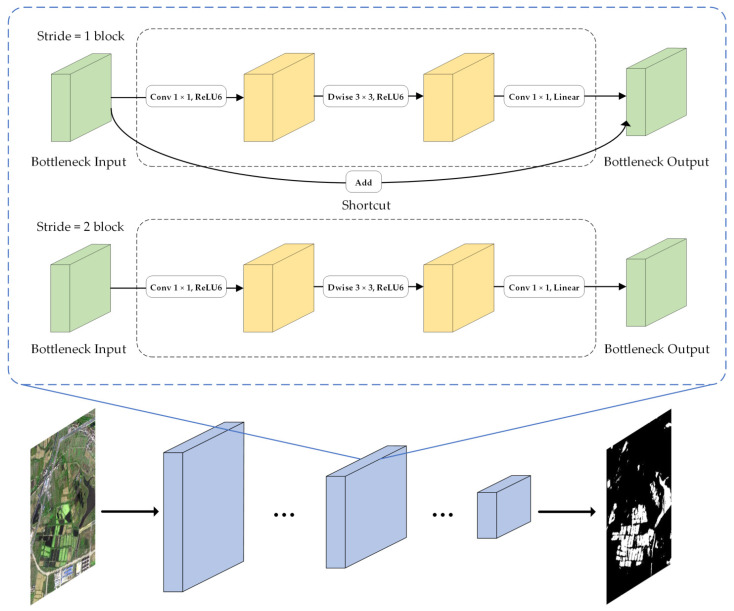
Network bottleneck structure of MobileNetV2.

MobileNetV2 is an improvement of MobileNetV1 and a lightweight CNN. Similar to MobileNetV1, MobileNetV2 also uses full convolution to make the model adapt to images of different sizes. Moreover, ReLU6 (maximum output is 6) is used as an activation function to make the model more robust under low-precision calculation [58]. The difference is that the model innovatively adjusts the internal structure of the model, further improves the network structure performance and reduces the computational complexity based on the deep separable convolution. The main improved structures include linear bottlenecks and inverted residual block. Linear bottlenecks use a linear activation function to replace the ReLU activation function of the smaller output dimension layer in the network, which avoids the destruction of feature diversity and enhances the expression ability of the network. The inverted residual block introduces the shortcut structure of ResNet, which can directly transfer shallow feature information to the deep layer. On the one hand, it solves the degradation problem of gradient divergence in deep learning models; and on the other hand, it can reuse features [59]. Contrary to the traditional residual block structure, the inverted residual block enhances the gradient propagation by increasing the dimension first and then decreasing the dimension, which greatly reduces the information loss caused by the ascending and descending order. The main network bottleneck structure of MobileNetV2 is shown in Figure 7. To match the shortcut dimension, when stride is 1, the shortcut was used, and when stride is 2, no shortcut was used. The network layer operations are shown in Table 2, where h is the input height, w is the width of input, k is the number of input channels, t is the expansion coefficient, s is the step length, and l is the number of output channels.

### 3.3. Construction of a Water Body Extraction Model Based on MobileNetV2

#### 3.3.1. Hardware and Software Environment Configuration

The computer used in this experiment was equipped with a 3.6 GHz eight-core Inter Core i7-9700K CPU (Inter Co., Madison, IL, USA). The display card is 11 GB of NVIDIA GeForce GTX 1080 Ti (NVIDIA Co., Santa Clara, CA, USA) and 32 G memory bars. The operating system is Windows Server 2019 Datacenter (Microsoft Co., Redmond, WA, USA). Matlab (MathWorks Co., Natick, MA, USA) and ArcGIS 10.6 (ESRI Co., Redlands, CA, USA) software were applied for data processing. This neural network design framework is a Tensorflow (Google Brain, Cambridge, MA, USA) deep learning framework.

#### 3.3.2. Construction of the Water Body Extraction Sample Set

Sample annotation: In this paper, the GF-2 10,080×10,080 image size, Worldview-2 and UAV 13,440×10,080 image size high-resolution remote sensing images were segmented by the Mean shift algorithm, and adjacent pixels with similar spectral characteristics were combined into a segmentation block. Then, the representative water bodies in 3 remote sensing images were annotated by ArcGIS 10.6. The background pixel RGB value was (0, 0, 0), and the water body pixel RGB value was (255, 255, 255). The label results were converted to GeoTiff format, which is used as the standard for training label data and model accuracy verification of the machine learning model and deep learning model.

Sample data clipping: Since the image slices in the study area are large in scale and cannot be directly sent to the deep learning model for training, sample image segmentation was performed on the semantic segmentation dataset [60]. Considering the training speed, training accuracy of the model and integrity of the spatial structure characteristics of training samples [61], the image was divided into size of 224×224 pixels and converted into GeoTiff format.

Data enhancement and sample allocation: 1125 images and 1125 annotation data points from GF-2 are contained in the segmented sample data set and 1500 images and annotation data points from Worldview-2 and UAV are included. We performed data augmentation on these sample data, including random rotation and translation. Among them, 60% was used as the training set, 20% was used as the test set, and 20% was used as the validation data set, and they are used to detect the model accuracy, generalization ability and practical application value. The allocation ratio of training set, validation set and test set was selected by comparative analysis of multiple experiments.

#### 3.3.3. Deep Learning Water Body Extraction Model Training

In this paper, the deep learning network used the weighted cross entropy as the loss function, the regularization was set to 0.005, the batch processing scale was set to 10, the epoch number was 20, and the initial learning rate was 0.001. The loss function was used in deep learning to estimate the inconsistency between the predicted value and the real value. We chose the weighted cross entropy as the loss function, which can calculate the derivative of the loss function with respect to each weight in the neural network, so the weight of the model can be adjusted accordingly to minimize the loss function. The regularization was used to punish the model complexity in deep learning, so as to reduce the over-fitting problem on the training data set and improve the generalization ability of the model. The optimization method is the random gradient descent method of driving quantity, which can accelerate the convergence of the network. We referred to other commonly used thresholds and determined these parameters through several experiments. We also discussed the parameter sensitivity analysis of epoch number in Section 5.1.

### 3.4. Accuracy Evaluation Method

In this study, the results of artificial visual interpretation were used as the accuracy evaluation criteria of the model, and the accuracy of the results was evaluated by 4 evaluation indices: *Precision* rate, *Recall* rate, *F1-score* and *Kappa* coefficient. Water body extraction in this paper can be regarded as a dichotomy problem, with the results classified as water bodies or non-water bodies. For binary classification problems, according to the combination of real categories and model classification categories, samples can be divided into 4 cases : true positive (*TP*), false-positive (*FP*), true negative (*TN*) and false negative (*FN*), and the results can be calculated using a pixel-based confusion matrix [62]. The *Precision* rate is the ratio of the number of correctly classified positive samples to the number of all samples separated by the classifier, the *Recall* rate is the ratio of the number of correctly classified positive samples to the number of positive samples, and the *F1-score* is the harmonic average of *Precision* and *Recall*. The *Kappa* coefficient was used for the consistency test because it can better measure the classification accuracy. The calculation formulas of each accuracy evaluation index are as follows:

(3) $$\mathrm{Precision}=\frac{\mathrm{TP}}{\mathrm{TP}+\mathrm{FP}}$$

(4) $$\mathrm{Recall}=\frac{\mathrm{TP}}{\mathrm{TP}+\mathrm{FN}}$$

(5) $$F1-\mathrm{score}=2\times \frac{\mathrm{Precision}\times \mathrm{Recall}}{\mathrm{Precision}+\mathrm{Recall}}$$

where TP is the number of pixels correctly extracted as water bodies, FN is the number of pixels correctly extracted as non-water bodies, FP is the number of pixels wrongly divided into non-water bodies, and TN is the number of pixels wrongly divided into non-water bodies.

(6) $$\mathrm{Kappa}=\frac{N\sum_{i=1}^{r}x_{\mathrm{ii}}-\sum_{i=1}^{r}\left(x_{i+}x_{+i}\right)}{N^{2}-\sum_{i=1}^{r}(x_{i+}x_{+i})}$$

where r is the total number of categories in the confusion matrix, N is the total number of pixels used for precision evaluation, $x_{\mathrm{ii}}$ is the total number of pixels correctly extracted in the confusion matrix, and $x_{i+}$ and $x_{+i}$ are the total number of pixels for each row and column of the confusion matrix, respectively.

## 4. Results

### 4.1. Comparison of Water Body Extraction Model Results

**Figure 8:**
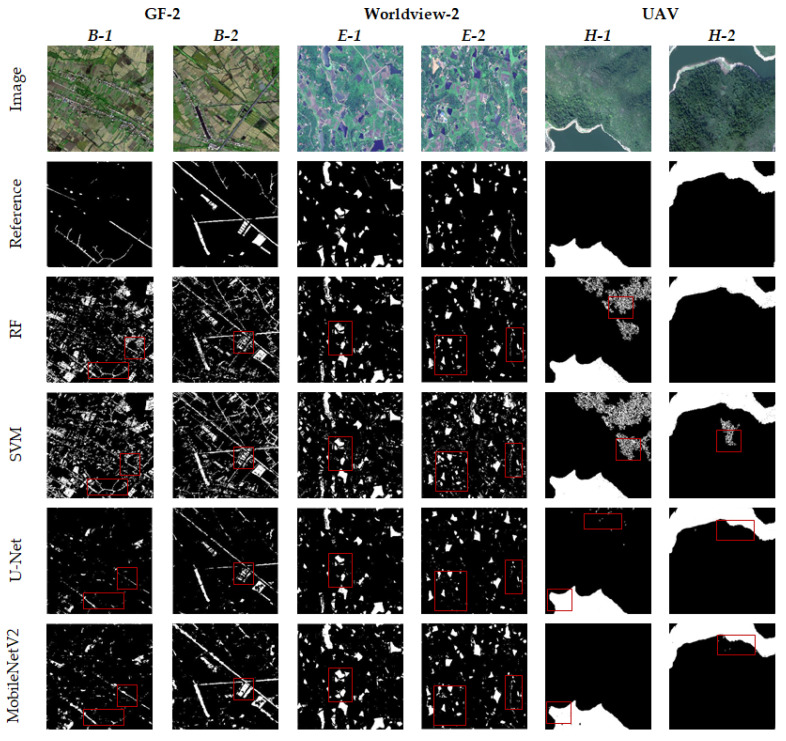
Comparison of water body extraction results by different models.

The machine learning models of SVM and RF and the deep learning models of U-Net and MobileNetV2 were applied to extract the water bodies. The overall comparison of the water body extraction results of different high-resolution test images is shown in Figure 8 (in the reference and results figures, white indicates water bodies and black indicates non-water bodies). Among them, GF-2 *B*-1, GF-2 *B*-2, Worldview-2 *E*-1, and Worldview-2 *E*-2 are plain areas, mainly with township buildings, irrigation paddy fields, ditches, broken lakes and slender rivers, and a considerable part of small-area water bodies are distributed around the water system. UAV *H*-1 and UAV *H*-2 are mountainous areas and mainly composed of large-scale rivers, vegetation and mountains. In the image, vegetation and mountain areas have obvious shadow areas.

Due to the pixel-oriented image segmentation method, the results of water body extraction may have scattered isolated pixels and holes, which is related to numerical anomalies of the image itself or differences between the models. In this paper, the ‘salt and pepper effect’ phenomenon of the extraction results was quantitatively counted. Through the application of morphological knowledge [63], isolated pixels less than a certain threshold are erased and holes less than a certain threshold are filled, which can improve the influence of noise. After several experiments, we deemed it appropriate to select the 10-pixel area as the threshold value, which can remove some noise points and holes in the result diagram, preserve more complete internal information of water body, and further improve the accuracy of water body extraction [64].

As shown in Figure 8, there is a big difference in the extraction of small water bodies in GF-2 and WorldView-2 images. The SVM model can be used to extract the vast majority of water bodies in each region, although a large degree of false extraction of shadows, farmlands and building intensity was observed and very large noise interference remained after noise treatment (GF-2 *B*-1 and Worldview-2 *E*-2). The overall effect of the RF model was better than that of the SVM, although a certain degree of error remains at the edges of water bodies, broken water bodies and farmland. Although the machine learning model has certain misrepresentations, it had a good effect on the continuity reservation of slender rivers. The accuracy of the two machine learning models depends on the selection and construction of sample data sets. Selecting different samples will lead to a large difference in the extraction results. The results of small water bodies extracted by the deep learning method in complex scenes were generally good, the effects of certain buildings, farmland and shadows were effectively removed, and the water area was extracted more accurately. Among them, the extraction effect of the U-Net model is slightly better than that of the machine learning method, which can accurately distinguish water bodies, farmlands, vegetation, shadows and buildings. However, it was easy to miss or break the flow in the extraction of broken lakes and slender rivers, and the boundary was relatively broken (GF-2 *B*-1, Worldview-2 *E*-1 and Worldview-2 *E*-2). The MobileNetV2 model achieved the best results in the boundary extraction of broken water bodies, ditches and slender rivers. At the same time, it was not affected by large areas of irrigated farmland, shadows and buildings, although a certain degree of leakage in some intact water bodies was observed (GF-2 *B*-2).

In the large-scale water body extraction of UAV test images, each model achieved good extraction results, but the extraction results of the machine learning model still have a certain degree of ‘salt and pepper noise’. The U-Net model in the deep learning model can better ensure the internal integrity of large water bodies, although a large degree of omission was observed in its boundary compared with the MobileNetV2 model. The MobileNetV2 model can better extract large-area water bodies, and the boundary was complete. Moreover, although the influence of vegetation shadows and mountain shadows on the extraction results was removed, some small holes will appear in the water bodies. In addition, the water areas extracted by manual visual interpretation, the SVM model, the RF model, the U-Net model, and the MobileNetV2 model were 7.25 hm^2^, 9.59 hm^2^, 8.49 hm^2^, 7.06 hm^2^, and 7.26 hm^2^, respectively. Compared with other models, the water area extracted by the MobileNetV2 model was closer to the real interpretation results, and the error was only 0.1%.

### 4.2. Accuracy Evaluation of Water Body Extraction Models

Taking the manually interpreted multisource and high-resolution remote sensing image as the standard image, the accuracy of the MobileNetV2 model for extracting water bodies was compared to that of the other three models, as shown in Table 3. The results show that in the three-view test images, the highest *F1-score* and *Kappa* coefficients were obtained for the MobileNetV2 model, which were 0.75 and 0.72, 0.86 and 0.85, 0.98 and 0.98. Compared with the subhigh-precision model, the *F1-score* and *Kappa* coefficients of the MobileNetV2 model increased by 11% and 13.7% on average, which is mainly because the MobileNetV2 model is far better than the other models in terms of shadow removal, small-area complex water body extraction and extraction integrity of each water body boundary. It can learn deeper spatial relationships, textures, shapes and other intrinsic characteristics of ground objects to more effectively distinguish water bodies and other similar spectral features and can adapt to the extraction of water bodies in complex scenes of different high-resolution images.

### 4.3. Water Body Extraction Generalization Verification of the MobileNetV2 Model

To study the spatial–temporal generalization and practical application value of the model, this paper selected other regions of the same county-level city in the three images as the model application generalization verification data set and performed radiation correction, geometric correction and image clipping on the generalization verification image. The GF-2 image with the image size of 3360×3360 pixels (area *C*) was divided into 225 images, and the Worldview-2 image with the image size of 4480×3360 pixels (area *F*) and the UAV orthophoto (area *I*) were divided into 300 images, and annotation data with the image size of 224 × 224 pixels were set as the generalization verification data sets. Based on the MobileNetV2 model and the training data from three individual sensors, the generalized validation datasets of the corresponding sensors were tested.

**Figure 9:**
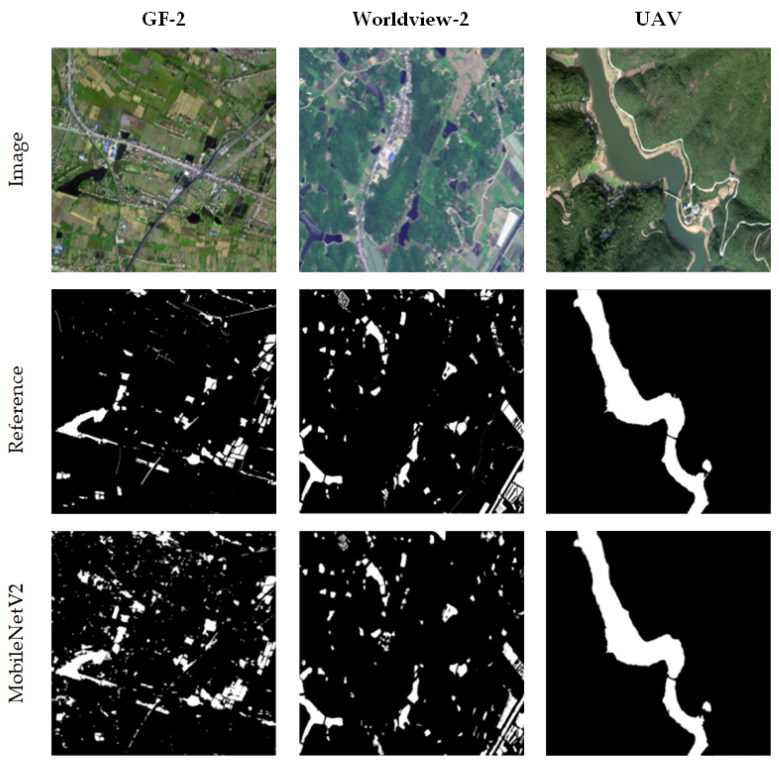
Comparison of the generalization verification results of MobileNetV2.

The generalization test extraction results are shown in Figure 9. There are some farmlands, irrigation ditches, broken lakes and slender rivers that are more difficult to distinguish between in the GF-2 and Worldview-2 generalization verification zones than in the training zone and the test zone. The UAV orthophoto generalization verification zone also shows increases in artificial construction facilities, such as bridges, and the river radians are also more curved, causing great difficulties in actual water body investigations. The MobileNetV2 model still maintained a good extraction effect in the above three generalization verification images, especially for large water bodies with large radians. The interior and boundary of the water body are relatively intact, and the discontinuous small area water body can also be better extracted. However, there are also obvious problems. The extraction results of the GF-2 generalization verification area had a certain degree of ‘salt and pepper noise’. The construction facilities adjacent to the water body were easily mistakenly identified as water bodies in the Worldview-2 generalization verification area. The slender and broken water bodies in some areas had more missing extraction, which may be due to the small sample of such water bodies in the process of model training. Table 4 shows the generalization verification accuracy of different high-resolution images. Experiments show that MobileNetV2 also has good scalability in multisource generalization verification images, and the *F1-score* and *Kappa* coefficients were still higher than those of the other three models in the test area, which has high engineering application value.

## 5. Discussion

### 5.1. Parameter Sensitivity Analysis

**Figure 10:**
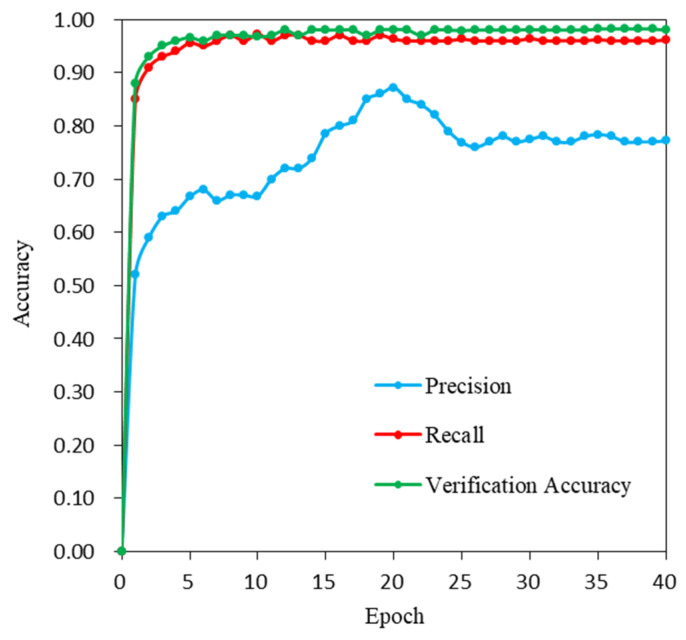
Epoch number sensitivity analysis.

This paper analyzes the sensitivity of the data training and verification accuracy of the MobileNetV2 network under different epochs, as shown in Figure 10. The results show that when the number of epochs was five, the model *Precision* rate was 66.7%, the verification accuracy was 96.6%, and the *Recall* rate was 95.6%. As the epoch number increased, the model verification accuracy increased. When the number of epochs reached 20, the *Precision* rate of the model was the highest at 87.2%. However, the accuracy decreased as the epoch number increased, indicating that more or fewer epochs are not conducive to the task of semantic image segmentation. With a low number of epochs, the model training will lose a large number of image features, and with an excessive number of epochs, overfitting of the model may occur. The accuracy of the model was higher only when the number of epochs was moderate, which is beneficial to obtaining fine and accurate extraction results.

### 5.2. Efficiency Comparison of the Deep Learning Models

The two deep learning models were compared to determine the efficiency of the water body extraction model. According to Formulas (1)–(2), the *Params* (number of parameters) and *FLOPs* of the deep learning model were calculated, and their average training times are shown in Table 5. The *average training time* of the MobileNetV2 model was much shorter than that of the U-Net model, which took approximately 645 s, because the number of parameters used and the computational resources occupied by the MobileNetV2 model were more than two times less than those of the U-Net model, which greatly improves the efficiency of the water body extraction model to achieve rapid and convenient extraction of complex water bodies in high-resolution images.

### 5.3. Influence of Spatial Resolution Change on the Accuracy of Water Body Extraction by the MobileNetV2 Model

To verify the influence of different spatial resolutions on the accuracy of water body extraction by the MobileNetV2 model, we grouped the training data sets of GF-2 (1 m), Worldview-2 (0.5 m) and UAV (0.2 m) images into four combinations and input them into the MobileNetV2 model for training, and then tested the generalization images of different sensors used in Figure 9. The generalized test results without any combination of sensor training data, in Table 4, were applied as a benchmark to calculate the changes of the accuracies for different combinations. The results of these changes are shown in Table 6. The results show that the model training with different spatial resolution images has a signification impact on the water body extraction. The different spatial resolution images indicate diverse complexities of background and water body samples, which may lead to the lack of feature similarities between the test images from individual sensor and the training images combined by multiple sensors. For example, when training the MobileNetV2 model using only the lower spatial resolution training dataset I, the water body extraction accuracy of the UAV generalization test images decreased significantly, with *F1-socore* and *Kappa* reduced by 0.46 and 0.56, respectively. Moreover, when the training data involved higher spatial resolution images, the MobileNetV2 model improved the accuracy of water body extraction from generalization test images of lower spatial resolution to varying degrees. The *F1-score* and *Kappa* with the training dataset II (combining the GF-2 with UAV images) were 0.08 and 0.09 higher, respectively, than those with the training dataset I (combining the Worldview-2 and GF-2 images). The *F1-score* and *Kappa* with the training dataset III (combing the Worldview-2 and UAV images) were 0.03 and 0.03 higher, respectively, than those with the training dataset of only the Worldview-2 images.

It is worth noting that with the training dataset IV, which was composed of these three different spatial resolution images, the *F1-score* and *Kappa* of the MobileNetV2 model on the generalization test images of GF-2 and Worldview-2 improved 0.04 and 0.04, 0.06 and 0.06, respectively. It indicates that the training data, combining relatively low spatial resolution images with high-spatial-resolution images, is beneficial for improving the accuracy of water body extraction from low spatial resolution test images. In addition, compared with the classification results with training data of only UAV images, the MobileNetV2 model, using any combination training data from UAV and other sensors, cannot improve the water body extraction accuracy for the UAV generalization test images. It indicates that training data by adding lower spatial resolution images may reduce the accuracy of water body extraction from higher spatial resolution images from the MobileNetV2 model.

### 5.4. Extraction Error Analysis of Mixed Water Bodies and Small Area Water Bodies

In this paper, MobileNetV2 was used to extract complex water bodies from three different high-spatial-resolution remote sensing images, and the accuracy was compared with that of the SVM, RF and U-Net models. MobileNetV2 shows greater advantages in efficiency and accuracy than other models and has a good overall effect in the three-view test images with strong robustness, which can be further applied to the actual water body investigation task. However, in complex environments where water bodies and other ground objects are mixed together, such as irrigated farmland with crops, edges of ditches with vegetation, and eutrophic water bodies, these models have large misclassifications and missed classifications. We analyzed the reasons for the low-precision extraction errors of the mixed water bodies and the broken, narrow and small-area water bodies.

The decrease in spatial resolution may lead to insufficient feature extraction of mixed water and small-area water. The three images in this paper come from different sensors and different spatial resolutions. Figure 9 shows that images with different spatial resolutions show very different extraction results. The MobileNetV2 model has the best extraction effect on UAV generalization test images, which may be related to the high-spatial-resolution of UAV images, which clearly display the boundary, texture and shape of water and other mixed ground objects so that the model can clearly extract and distinguish the characteristics of different ground objects during training. However, on the Worldview-2 generalization test image with slightly lower spatial resolution than the UAV image, MobileNetV2 mistakenly identified multiple farmlands and artificial facilities as water bodies and lacked complete extraction of small-area eutrophic water bodies and farmland irrigation water bodies. On GF-2 images with lower spatial resolution, not only farmland but also large dark vegetation around lakes were misidentified as water by the MobileNetV2 model. As seen from Table 6, these misclassifications and missing classifications of mixed water and small water areas reduced the *F1-score* and *Kappa* of the MobileNetV2 model from 0.82 and 0.81 for Worldview-2 to 0.64 and 0.61 for GF-2, respectively. The reason may be that as the spatial resolution of the image increases, the area of a single pixel also gradually increases, which leads to lost information for small water bodies with relatively broken shapes and narrow boundaries and slender ditches; moreover, the spatial structure of the image changes [65], resulting in the problem of mixed pixels, which leads to insufficient feature extraction for each mixed water body and small water body when the model is trained in complex scenes. The training data of higher resolution images may alleviate the problem of incomplete feature extraction caused by mixed pixels. As shown in Table 6, with training dataset IV, after adding Worldview-2 and UAV training data, which have a higher spatial resolution than GF-2 image training data, the MoblieNetV2 model improves the accuracy of water extraction in the GF-2 and Worldview-2 generalization test images.

The performance of the model itself has a great influence on the water body extraction results. The SVM, RF, U-Net and MobileNetV2 models used in this paper have different structures and performances. SVM and RF are the classic models of machine learning, and their advantages and disadvantages are obvious. The SVM model can deal with high-dimensional data well, but it is sensitive to missing data. Therefore, water bodies with obvious characteristics can be extracted in each image, although the accuracy cannot be guaranteed for complex water bodies. The RF model has a strong antinoise ability and can deal with missing data. The extraction effect in each image is relatively stable, although in complex scenes, it will also fall into overfitting, resulting in reduced model test accuracy. As deep learning algorithms, U-Net and MobileNetV2 have significant advantages for complex, large-scale data processing. However, under the complex background of mixed ground objects and dark objects with more similar spectral characteristics, insufficient feature extraction will occur. It is necessary to further improve or innovate the network structure of feature extraction to enhance the feature extraction ability. In the future, by combining GIS spatial analysis technology or other data sources, the recognition ability of mixed water bodies and small-area water bodies in complex scenes could be enhanced.

## 6. Conclusions

In this paper, we applied the lightweight network MobileNetV2 to extract the water bodies from GF-2, Worldview-2 and UAV images. The results show that the *F1-score* and *Kappa* of the water body extraction results of the MobileNetV2 model were higher than those of the SVM, RF and U-Net models, which were 0.75 and 0.72 for GF-2, 0.86 and 0.85 for Worldview-2, 0.98 and 0.98 for UAV, respectively. Our model improves the difficulty of feature extraction in traditional methods and is less affected by interferential features such as irrigated farmland, shadows and buildings. Moreover, this method uses much less parameters, calculation and training time than U-Net model, which greatly improved the efficiency of algorithm. In order to verify the generalization ability of the MobileNetV2 model, we respectively selected the areas with more cultivated land, building facilities, shadows and complex water bodies from these three sensor images. The results show that our model still maintains high extraction accuracies, and the *F1-score* and *Kappa* were 0.64 and 0.61 for GF-2, 0.82 and 0.81 for Worldview-2, 0.98 and 0.98 for UAV, respectively. Additionally, we analyzed the influence of different spatial resolution images from multiple sensors. It reveals that MobileNetV2 model could achieve higher accuracy in water body extraction by training with only the higher spatial resolution sample data, or training with the combination of lower and higher spatial resolution images, according to the existed remote sensing images. For the purpose of applying MobileNetV2 model to mixed and small area water bodies, we will further improve the feature extraction structure in this network and combine it with GIS spatial analysis technology.

## Figures and Tables

**Table 1:** Overview of the study area and data sources.

Area	Sensor	Image Date	Spatial Resolution	Image Size
*A*–*C*	GF-2	2017.9.30	1 m	10,080 × 10,080
*D*–*F*	Worldview-2	2016.4.30	0.5 m	13,440 × 10,080
*G*–*I*	UAV	2019.7.30	0.2 m	13,440 × 10,080

**Table 2:** Input and output of network layers in MobileNetV2.

Input	Operation	Output
h×w×k	1×1 Conv2d, ReLU6	h×w×(tk)
h×w×(tk)	3×3Dwises=s, ReLU6	$\frac{h}{s}\times \frac{w}{s}\times (\mathrm{tk})$
$\frac{h}{s}\times \frac{w}{s}\times (\mathrm{tk})$	Linear 1×1 Conv2d	$\frac{h}{s}\times \frac{w}{s}\times l$

**Table 3:** Accuracy evaluation of water body extraction results.

Sensor	Model	*Precision*	*Recall*	*F1-Score*	*Kappa*
GF-2	RF	49.6%	80.9%	0.61	0.57
	SVM	34.3%	87.9%	0.49	0.44
	U-Net	58.1%	69.5%	0.63	0.59
	MoblieNetV2	66.6%	85.5%	0.75	0.72
Worldview-2	RF	69.3%	90.7%	0.79	0.74
	SVM	53.9%	90.4%	0.68	0.65
	U-Net	88.1%	67.6%	0.77	0.75
	MoblieNetV2	77.2%	96.3%	0.86	0.85
UAV	RF	82.5%	96.2%	0.89	0.87
	SVM	72.5%	97.6%	0.83	0.81
	U-Net	99.3%	79.7%	0.88	0.87
	MoblieNetV2	97.3%	99.5%	0.98	0.98

**Table 4:** Accuracy evaluation of the generalization verification results of MobileNetV2.

Sensor	*Precision*	*Recall*	*F1-Score*	*Kappa*
GF-2	51.4%	84.9%	0.64	0.61
Worldview-2	80.2%	84.7%	0.82	0.81
UAV	96.3%	99.5%	0.98	0.98

**Table 5:** Efficiency of different deep learning models.

Model	*Params* (Million)	*FLOPs* (Million)	*Average Training Time* (Second)
U-Net	7.76	685	903
MobileNetV2	3.40	300	645

**Table 6:** Comparison of the effects of different spatial resolution training dataset combinations based on MobileNetV2 and the accuracy of different sensor generalization test images. Values reported are differences from the baseline model results reported in Table 4.

Mode	Sensor	*Precision*	*Recall*	*F1-Score*	*Kappa*
Training I: GF-2 and Worldview-2	GF-2	−10%	6.2%	−0.07	−0.08
	Worldview-2	3.3%	3.7%	0.04	0.04
	UAV	−61.5%	−0.1%	−0.46	−0.56
Training II: GF-2 and UAV	GF-2	1.3%	−0.1%	0.01	0.01
	Worldview-2	−7.2%	−20.6%	−0.14	−0.15
	UAV	−0.5%	−1.6%	−0.01	−0.02
Training III: Worldview-2 and UAV	GF-2	−0.9%	−15.2%	−0.05	−0.06
	Worldview-2	11.4%	−4.6%	0.03	0.03
	UAV	1.3%	−2.3%	−0.01	−0.01
Training IV: GF-2 and Worldview-2 and UAV	GF-2	5.2%	−1.1%	0.04	0.04
	Worldview-2	12.7%	−1.3%	0.06	0.06
	UAV	−1.3%	−1.2%	−0.01	−0.02

## Data Availability

The data presented in this study are openly available in FigShare at https://doi.org/10.6084/m9.figshare.16859593.v1 (accessed 22 October 2021).
